# Fabrication of Fibrin/Polyvinyl Alcohol Scaffolds for Skin Tissue Engineering via Emulsion Templating

**DOI:** 10.3390/polym15051151

**Published:** 2023-02-24

**Authors:** Guoying Zhou, Jiayan Zhu, Catriona Inverarity, Yifeng Fang, Zhao Zhang, Hua Ye, Zhanfeng Cui, Linh Nguyen, Haitong Wan, Julian F. Dye

**Affiliations:** 1 College of Life Sciences, Zhejiang Chinese Medical University, 548 Binwen Road, Hangzhou 310053, China; 2 Institute of Biomedical Engineering, Department of Engineering Science, University of Oxford, Oxford OX1 2JD, UK; 3 Department of Life & Health Sciences, The Open University, Milton Keynes MK7 6AA, UK; 4 Division of Biomaterials and Tissue Engineering, Eastman Dental Institute, University College London, Royal Free Hospital, Pond Street, London NW3 2QG, UK

**Keywords:** fibrin, PVA, skin scaffold, emulsion templating, wound healing, skin tissue engineering

## Abstract

In the search for a novel and scalable skin scaffold for wound healing and tissue regeneration, we fabricated a class of fibrin/polyvinyl alcohol (PVA) scaffolds using an emulsion templating method. The fibrin/PVA scaffolds were formed by enzymatic coagulation of fibrinogen with thrombin in the presence of PVA as a bulking agent and an emulsion phase as the porogen, with glutaraldehyde as the cross-linking agent. After freeze drying, the scaffolds were characterized and evaluated for biocompatibility and efficacy of dermal reconstruction. SEM analysis showed that the formed scaffolds had interconnected porous structures (average pore size e was around 330 µm) and preserved the nano-scale fibrous architecture of the fibrin. Mechanical testing showed that the scaffolds’ ultimate tensile strength was around 0.12 MPa with an elongation of around 50%. The proteolytic degradation of scaffolds could be controlled over a wide range by varying the type or degree of cross-linking and by fibrin/PVA composition. Assessment of cytocompatibility by human mesenchymal stem cell (MSC) proliferation assays shows that MSC can attach, penetrate, and proliferate into the fibrin/PVA scaffolds with an elongated and stretched morphology. The efficacy of scaffolds for tissue reconstruction was evaluated in a murine full-thickness skin excision defect model. The scaffolds were integrated and resorbed without inflammatory infiltration and, compared to control wounds, promoted deeper neodermal formation, greater collagen fiber deposition, facilitated angiogenesis, and significantly accelerated wound healing and epithelial closure. The experimental data showed that the fabricated fibrin/PVA scaffolds are promising for skin repair and skin tissue engineering.

## 1. Introduction

Skin is an important organ of the human body, acting not only as the first line of defense against external insults but also for thermoregulation, fluid homeostasis, self-healing, sensation, and appearance [1]. Critical-sized damage or loss of skin breaches the skin’s barrier functions and has serious consequences [2]. According to the World Health Organization, nearly 300,000 people die each year from scalds and burns, and 11 million of patients suffer extensive skin loss from accidents that often result in costly treatment and even death [3,4,5,6]. The conventional way to treat most full-thickness wounds by using autologous split-thickness skin grafting is constrained by inevitable scar formation, which occurs in the absence of dermal tissue, and the need for creating a donor site wound, which can be more painful than the original wound and be prone to infection [7]. Synthetic skin substitutes from tissue engineering are emerging as a promising alternative approach [8]. Particularly in the cases of large-area skin loss injuries, which are mostly caused by burns and severe trauma, as well as ulcerative skin loss in chronic wounds, such as diabetic foot ulcers, venous leg ulcers, and pressure ulcers, the development of bio-intelligent scaffolds for tissue reconstruction as skin substitutes could provide a reconstructive solution [9,10,11]. A successful skin tissue scaffold should meet the following requirements: wound biocompatibility, cell adhesivity, porosity for tissue cell ingrowth and proliferation, suitable mechanical property, and biodegradability [1,12]. Many types of skin replacement have been developed in recent decades [1], fabricated either from natural purified polymers, such as collagen, gelatine (denatured collagen), fibrin, glycosaminoglycans, chitosan, alginate, or from synthetic polymers, including poly(glycolic acid) (PGA), poly(L lactic acid) (PLLA), polycaprolactone (PCL), polyethylene glycol (PEG), and polyvinyl alcohol (PVA) [13,14,15,16].

Fibrin is an insoluble fibrous web of proteins formed by the conversion of soluble precursor molecular fibrinogen by the serine protease thrombin [17,18]. It has been reported as a promising candidate for skin scaffold fabrication due to its good pro-angiogenic effects, excellent biocompatibility and biodegradability, as well as tunable physico-chemical features [19,20,21]. However, the use of fibrin in skin tissue engineering has been limited since the hydrogel and scaffolds formed from fibrin have low mechanical properties, rapid degradation rates, and poor manipulability in clinical applications [22,23]. In addition, human GMP-graded fibrin is expensive, and the resources are quite limited. Therefore, fibrin is often combined with other polymers, such as polyethylene oxide [24], polycaprolactone [25], and chitosan [26], to overcome the limitation of fibrin. Polyvinyl alcohol (PVA), as a water-soluble synthetic polymer, has been widely used in biomedical fields because of its non-toxic, excellent hydrophilicity, and degradable properties [27]. More importantly, PVA has the property of cryogellation, and PVA cryogels have been exploited for biomedical applications [28,29]. Previous studies have shown that the incorporation of PVA can not only improve the mechanical strength of protein materials, such as fibrin, but may reduce the cost of scaffolds [30,31]. Fibrin is an extracellular matrix protein that can confer proangiogenic and cyto-conductive effects that the PVA scaffold itself lacks [32]. Therefore, the combination of fibrin and PVA will be expected to improve the outcomes for scalable skin scaffold manufacture. Yang et al. fabricated a series of konjac glucomannan/polyvinyl alcohol (KGM/PVA) composites followed by fibrinogen adsorption, which was found to enhance skin healing properties [33]. In another study by Xu et al., granule lyophilized platelet-rich fibrin was incorporated into PVA hydrogel, and the fibrin/PVA hydrogel exhibited significant effects in accelerating wound closure in an acute full-thickness dorsal skin wound model [34].

Recently, the emulsion templating method has attracted much attention for the manufacture of various high-porosity polymer scaffolds due to the possibility of controlling porosity (up to 90% of material volume), high interconnectivity, high diffusion properties and cost-effectiveness [35,36,37]. This method is based on the preparation of at least two immiscible solutions as emulsions, in which one phase (inner phase, dispersed phase) is dispersed in another phase (continuous phase, outer phase), followed by solidification of the continuous phase of the emulsion. During this process, the droplets of the dispersed phase behave as templates that can be removed after solidification to obtain a porous matrix. These polyphase emulsion systems can be either water-in-oil (w/o) or oil-in-water (o/w), depending on the location of the lipophilic and hydrophilic phases [38]. In our previous studies, we developed an emulsion templating method to fabricate micro-porous and nano-fibrous native protein scaffolds and demonstrated the feasibility of this method for non-cytotoxic, cyto-compatible tissue matrices [39]. However, the use of PVA in emulsion templating to create a composite structure has, to our knowledge, not been reported before.

In the present study, we sought to investigate whether combining the physical structure, which could be achieved by a PVA cryogel, with the cytoadhesive and pro-vascular properties of fibrin, would enhance the biological integration and organization of neo-tissue formation. Porous fibrin and fibrin/PVA scaffolds were fabricated by using a previously developed emulsion templating method applied to these formulations [39]. The critical design parameters of scaffolds were characterized, specifically, structure and pore size by SEM, the mechanical properties by tensile testing, and proteolytic stability by trypsin degradation rate. The cytocompatibility of the scaffolds was studied by cell proliferation using MSCs, and tissue regenerative potential was evaluated in a full-thickness skin excision model. The results are reported herein.

## 2. Materials and Methods

### 2.1. Fibrin/PVA Scaffold Manufacture

**Figure 1:**
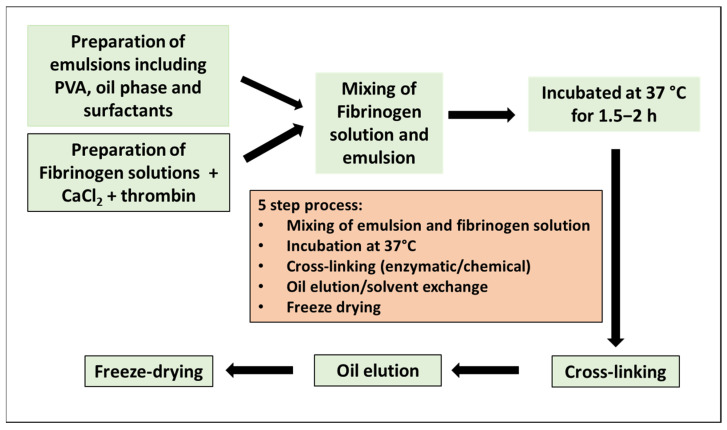
Schematic flow chart for Fibrin/PVA scaffold manufacturing.

An amount of 2% (*w*/*v*) bovine plasma fibrinogen (FNG, Sigma, Manchester, UK) and 5% (*w*/*v*) polyvinyl alcohol (PVA, Mw 205 kD, Sigma) was prepared in 25 mM MES/150 mM NaCl (pH 7.4) and pre-warmed at 37 °C before making the scaffolds. Emulsion mixtures comprising PVA, decane (Sigma), surfactant Triton CG110 (Sigma), and aqueous buffer 25 mM MES/150 mM NaCl (pH 7.40) were mixed in a 7 mL Bijou tube (Thermo Fisher scientific, Oxford, UK) using a pulsatile method, allowing the emulsion phase to settle and accumulate underneath the decane phase. A point should be reached when the mixture is inverted, the viscosity is increased, and the full O/W phase is established. The mixtures were blended for an additional 30 s to ensure even mixing. For the preparation of the scaffold, 1 M CaCl_2_ with a defined volume of FNG solution was added to another 7 mL Bijou tube, followed by the addition of thrombin and gently mixed by swirling for 10 s. Afterward, the prepared emulsion mixtures were added and blended for a further 30 s. The coagulation mixture was then poured into a pre-labeled casting tray and incubated at 37 °C for 1 h. After incubation, the scaffolds were cross-linked by 0.2% (*v*/*v*) glutaraldehyde (Sigma) in 20% MES/80% Ethanol for 4 h. To investigate the effects of cross-linking degree on mechanical and degradation properties, the scaffolds were cross-linked by 0.05%, 0.2%, 0.5%, or 1% (*w*/*v*) glutaraldehyde (25% *w*/*v*), respectively, and the one without glutaraldehyde as a control. The cross-linked structures were subsequently stabilized by adding 0.1% NaBH_4_ as a reducing agent. During the degradation experiments, N-ethyl-N′-[3-dimethylaminopropyl] carbodiimide/N-hydroxy succinimide (EDC/NHS) (200 mM of both EDC and NHS) was used as an alternative cross-linking agent. Finally, the scaffolds were washed with distilled water and freeze-dried using a pilot scale shelf-controlled programmable unit (Genesis II, BPS, Winchester, UK) with software control. The scaffolds with different compositions were labeled as FNGaPVAb, where a and b represented the concentrations (*w*/*v*) of FNG and PVA, respectively. The flow chart of the scaffold manufacturing is shown in Figure 1.

### 2.2. Fourier Transform-Infrared Spectroscopy (FT-IR) Analysis

Raw materials of PVA, freeze-dried FNG1, and FNG1PVA1 scaffolds were mixed with KBr separately and pressed at 10 MPa into prepared disks of 10 mm diameter. The prepared samples were then recorded using FT-IR (Nicolet IS50 Fourier transform-infrared spectrometer, Waltham, MA, USA) in the range of 400–4000 cm^−1^ wave number.

### 2.3. Microstructure and Pore Size of Fibrin/PVA Scaffold

Scaffolds were cut using a scalpel along their cross-sections, gold splutter-coated, and the microarchitectures were observed by scanning electron microscopy (SEM, Carl Zeiss Evo LS15 VP-Scanning Electron Microscope SE, BSE, VPSE, EPSE detectors) at an accelerating voltage of 15 kV. Three images were taken per scaffold, and the diameters of 20 random pores were averaged out to calculate the estimated mean pore size of each scaffold.

### 2.4. Mechanical Properties of Fibrin/PVA Scaffold

The fibrin/PVA scaffolds were cut into strips in the size of 10 mm × 50 mm × T, where T is the variable thickness of the scaffold. The two ends of the strips were anchored into the jaws of the mechanical testing machine (Instron 5982 UTM, High Wycombe, UK) and stretched until the scaffold broke into two pieces. Ultimate tensile strength data for each scaffold were calculated from the stress–strain curves. Elongation of scaffolds was defined as the displacement at break/original length. Each sample was performed in triplicate.

### 2.5. Degradation Profile of Fibrin/PVA Scaffold

Scaffolds were cut into diameters of 8 mm using a punch biopsy and incubated in 0.25% Trypsin-EDTA (1×) (Thermo Fisher Scientific, Oxford, UK) at 37 °C, which was used as an enzymatic proteolysis assay to compare the degradation profiles of each scaffold, over a relatively short period of 1, 3, and 5 days, respectively. The trypsin solution was changed daily for up to 5 days. After each time point, the scaffolds were rinsed with distilled water and freeze-dried, and the dry mass of the scaffolds was recorded for calculation of the degradation rate. The fraction remaining was defined as dry weight after degradation/initial dry weight. Each sample was performed in triplicate.

### 2.6. Cell Experiments

#### 2.6.1. Cell Culture

Green Fluorescence Protein (GFP) cloned mesenchymal stem cells (MSC, kindly provided by Dr. James Liu, Department of Paediatrics and Adolescent Medicine, LKS Faculty of Medicine, The University of Hong Kong) were cultured in Dulbecco’s modified Eagle’s medium (DMEM 1.0 g/L of glucose; Gibco BRL, Gaithersburg, MD, USA) supplemented with 10% (*v*/*v*) fetal bovine serum (FBS; Gibco BRL) and 1% (*v*/*v*) penicillin-streptomycin (PS; Gibco BRL) at 37 °C in a humidified incubator aerated with a blend of air with 5% CO_2_.

#### 2.6.2. Cell Proliferation in Fibrin/PVA Scaffolds

For the cell proliferation experiment, the scaffolds were cut into diameters of 8 mm using a punch biopsy and placed into a 48-well tissue culture plate, sterilized with 70% ethanol for 15 min, and rinsed with sterile PBS twice, followed by rinsing with fresh DMEM medium once. A total of 0.1 mL of human bone marrow MSC cell suspension was added onto the scaffold at a concentration of 5 × 10^4^ cells/well in the 48-well plate and incubated for 30 min. Thereafter, 0.4 mL of 10% FBS-containing DMEM medium was added around the scaffold. The cells were then incubated for 1, 7, and 14 days at 37 °C in a 5% CO_2_/95% air incubator. Afterward, a cell-counting kit-8 (CCK-8) assay (Sigma) was applied to quantify the amount of metabolic active cells. Briefly, the scaffolds with cells were transferred to a new 48-well tissue culture plate (in order to remove any possible effect of cells attached to the tissue culture plate) and washed once with sterile PBS. Then, 300 µL of pre-warmed 1% FBS-containing DMEM with 30 µL of CCK-8 assay reagent (10:1) was added to each well and incubated at 37 °C for 4 h. Thereafter, 100 µL of supernatant in triplicate from each well was transferred to a 96-well plate, and the absorbance was measured at 450 nm in a microtiter plate reader.

#### 2.6.3. Cell Morphology on Fibrin/PVA Scaffolds

After culturing for 14 days, the cell-containing scaffolds were washed with PBS three times. The cells on the scaffold were fixed with 5% glutaraldehyde (Sigma, Manchester, UK) in PBS for 30 min at room temperature. After rinsing with distilled water three times, the samples were then dehydrated sequentially in 50, 70, 90, and 100% ethanol for 2 × 10 min, respectively. Afterward, the scaffolds were chemically dried by 2:1, 1:1, and 1:2 of ethanol/hexamethyldisilazane (HMDS, Sigma–Aldrich, UK) for 15 min exposure at each step, 100% HMDS for 3 × 15 min, and then air-dried for HMDS removal [40]. The scaffolds were sputter-coated with gold and examined by SEM (Carl Zeiss Evo LS15 VP-Scanning Electron Microscope with SE, BSE, VPSE, and EPSE detectors) at an accelerating voltage of 15 kV.

### 2.7. Establishment of Full-Thickness Skin Wound Model and Would Healing Evaluation

Male C57 mice at 25–30 g were purchased from Shanghai Slack Laboratory Animal Co., LTD and maintained under controlled temperature (20 ± 2 °C) and humidity (50 ± 10%). All experimental procedures were performed in accordance with local animal welfare laws and guidelines and were approved by the Animal Ethics Committee of Zhejiang Chinese Medical University (Approval No.: IACUC-20221031-23).

After two weeks of adaptive feeding, the mice fasted for 12 h, and a full-thickness skin wound model was established. Briefly, the mice were anesthetized with 3% pentobarbital sodium and then shaved. Afterward, a full-thickness skin wound with a diameter of 6 mm was created on the dorsal of mice, four wounds for each mouse. The mice with full-thickness skin defects were then randomly separated into four groups: the control group; FNG1 treated group; and FNG1PVA0.5 and FNG1PVA1 treated groups. The control group was not treated after skin defects, while mice in the FNG1, FNG1PVA0.5, and FNG1PVA1 groups were grafted with the corresponding scaffolds followed by being wrapped with 3 M Tegaderm film on the wound site. Digital photographs of the wound areas were taken on days 0, 5,10, and 14. The quantified wound area at each time point was determined using ImageJ software. The normalized wound area was calculated using the following formula: Normalized wound area (%) = A_d_/A_0_ × 100%, where A_d_ and A_0_ are the wound area on day 0 and day d after treatment.

### 2.8. Histological Staining Analysis

After C57 mice were sacrificed on day 14, the regenerated skin around the wounds was removed and fixed with 4% paraformaldehyde. Tissue samples were then embedded in paraffin and sectioned for hematoxylin and eosin staining (H&E) and Masson trichrome staining. The images were observed through a light microscope (Olympus, Tokyo, Japan).

### 2.9. Immunofluorescence Staining

Briefly, after deparaffinization, hydration, and antigen retrieval, the skin tissue sections were blocked with 5% BSA at room temperature for 1 h and then incubated with the primary antibodies rabbit anti-CD31(1:300, Servicebio, Wuhan, China) and mouse anti-α-SMA (1:200, Servicebio) (dissolved in 1% BSA) at 4 °C overnight. After washing with PBS, the sections were incubated with Cy3-conjugated goat anti-rabbit IgG (1:300, Servicebio) and Alexa Flour 488-conjugated goat anti-mouse IgG (1:400, Servicebio) at room temperature for 1 h. Afterward, DAPI (Servicebio) was added dropwise and incubated for 10 min. Finally, sections were captured by the virtual slide microscope (VS120-S6-W, OLYMPUS), and the analysis of CD31 and α-SMA positive staining cells was quantified using ImageJ software.

### 2.10. Statistics

All data are represented as mean values ± standard deviations (SD). Statistical analysis was performed using one-way ANOVA followed by post-hoc Tukey testing using origin software. The significance level was set as *p* < 0.05 and is indicated by an asterisk.

## 3. Results and Discussion

### 3.1. Scaffold Composition, Structure, and Porosity

**Figure 2:**
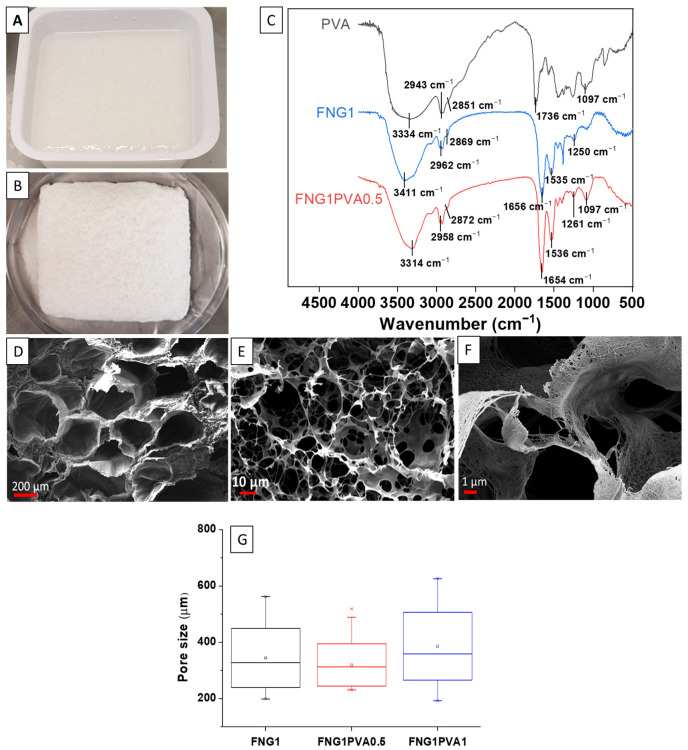
The photographs of the FNG1PVA0.5 scaffold before (**A**) and after (**B**) freeze drying. (**C**) FT-IR spectra of PVA, pure FNG scaffold (FNG1), and FNG1PVA0.5 scaffold. Scanning electron microscopy (SEM) characterization of FNG1PVA0.5 scaffolds with low and high magnification (**D**–**F**), as well as a quantitative pore size analysis (**G**) of the fibrin/PVA scaffolds from SEM images. Box—whisker diagrams indicate the 25th and 75th percentile, the median (dash, −), mean (square, □), and 1st and 99th percentiles (cross, ×), respectively. FNGaPVAb representing the concentrations of FNG and PVA for making the scaffold are a% and b%, respectively, while FNGa representing the concentration of FNG for making the scaffold is a% without PVA.

Figure 2A,B demonstrates the photographs of the FNG1PVA0.5 scaffold before (wet state) and after (dried state) freeze-drying, respectively. The FT-IR spectra of PVA, pure FNG scaffold (FNG1), and PVA-incorporated scaffold (FNG1PVA0.5) are presented in Figure 2C. The FT-IR spectrum of PVA showed characteristic absorption bands for O–H (3500~3200 cm^−1^), C–H (2943 and 2851 cm^−1^), and C=O (1736 cm^−1^) peaks. FT-IR spectra of FNG1 showed characteristic peaks for amide I C=O, amide II N-H, and amide III C–N stretching vibrations around 1656 cm^−1^, 1535 cm^−1^, and 1240 cm^−1^, respectively. The band in the region of 3411 cm^−1^ corresponds to the vibrations of the hydroxyl groups in FNG1. FNG1PVA0.5 scaffold showed the O–H stretching band of FNG1 at 3314 cm^−1^. However, this absorption band shifted slightly to the lower wavelength region. This strongly indicates the formation of new hydrogen bonds because hydrogen bond formation could make the density of the electron cloud average and reduce the frequency of stretching vibration, leading to the shift of the absorption peak to a lower wavelength. Similar hydrogen formation has been observed between PVA and other compounds by others [41,42,43,44]. Additionally, C–H stretching at around 2958 and 2872 cm^−1^ was found to enhance slightly, which might be due to the incorporation of PVA with abundant alkyl groups. Overall, the FT-IR data prove the successful incorporation of PVA in the FNG1PVA0.5 scaffold.

Figure 2D–F shows the representative SEM images of the porous FNG1PVA0.5 scaffold. Interconnected pores were found on the scaffold, as shown in Figure 2E. In addition, Figure 2F demonstrates a nano-scale fibrous architecture for the FNG1PVA0.5 scaffold in addition to the micro-scale porous structure. Moreover, a quantitative analysis of the pore size formed on the different scaffolds was summarized in Figure 2G. It was found that the median pore diameter was around 330 µm in all scaffolds. As expected, this did not vary much among scaffolds with different formulations because the structure of the porous scaffolds is mainly controlled by the fabrication process. However, it is notable that the FNG1PVA0.5 scaffold showed a more uniform pore size distribution (333 ± 78) compared to FNG1 (359 ± 109) and FNG1PVA1(446 ± 109) scaffold, indicating a more appropriate formulation for scaffold fabricating.

### 3.2. Mechanical Properties of Scaffold

**Figure 3:**
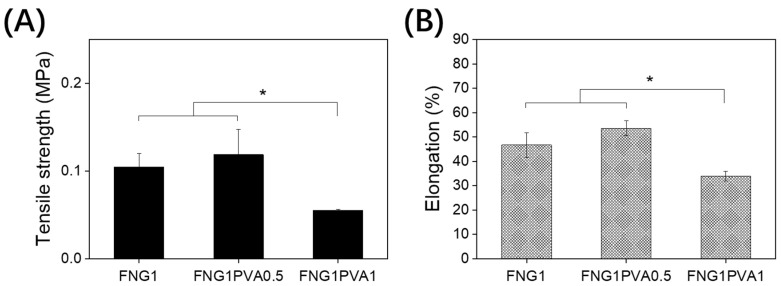
Mechanical property characterization of the fibrin/PVA scaffolds, including ultimate tensile strength (**A**) and elongation of scaffold at break (**B**). Ultimate tensile strength data for each scaffold were calculated from the stress–strain curves. Elongation of scaffold was defined as the displacement at break/original length. Each sample was performed in triplicate. Data represent mean ± SD, *n* = 3, * *p* < 0.05.

Mechanical properties of skin products are very important for scaffold manufacture, handling during surgery, and application post-surgery. The mechanical properties of the scaffolds were studied using a Tensiometer at a strain rate of 1 mm/min. The results were analyzed in terms of ultimate tensile strength, and the elongation of the scaffold at break is shown in Figure 3.

Figure 3A shows that FNG1 and FNG1PVA0.5 possessed ultimate tensile strength at around 0.12 Mpa, and FNG1PVA0.5 had slightly higher ultimate tensile strength than that of FNG1. By contrast, the FNG1PVA1 scaffold had a significantly lower ultimate tensile. The elongations of the scaffold at the break for FNG1, FNG1PVA0.5, and FNG1PVA1 (Figure 3B) were 46.7%, 53.6%, and 34.0%, respectively. This indicates that the incorporation of PVA in FNG/PVA scaffolds can produce modest but significant effects on the ultimate tensile strength and elongation of the scaffolds. As a result, a lower ratio (FNG1PVA0.5) of PVA incorporation can improve the mechanical properties, but contrary to expectation, the incorporation of PVA at a higher ratio (FNG1PVA1), however, reduces the mechanical properties of the product. This might be due to coacervation between fibrinogen and PVA at a high concentration [45]. Such a phase separation may interrupt the network of fibrin formation to some extent, leading to the non-uniformity of the formed scaffold and lowering of the mechanical properties. Although the tensile strength of the composite FNG1PVA0.5 was not increased significantly, the Young modulus of these materials is in the same range compared with other PVA cryogels and composites [46]. However, the manipulability of composite scaffolds was markedly improved both during scaffold fabrication processes and surgical handling practical benefit of PVA incorporation. This may be attributed to the effect of inter-penetrating PVA cryogel structure.

### 3.3. Enzymatic Degradation of Scaffold

**Figure 4:**
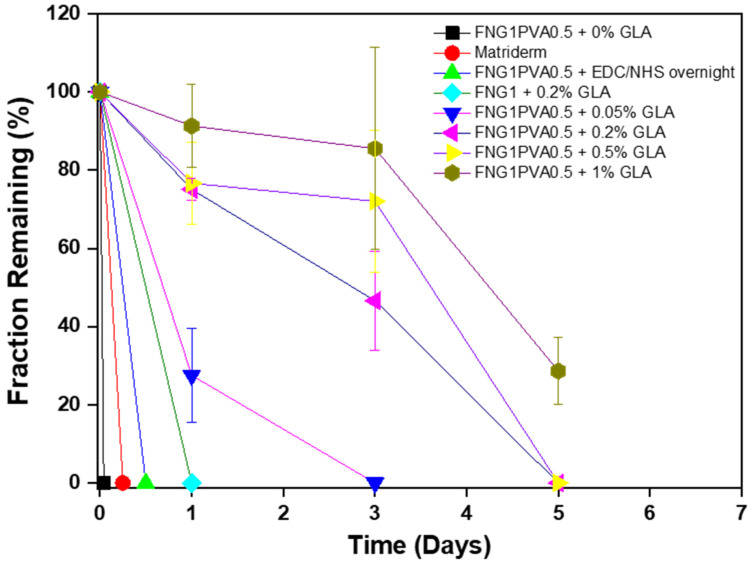
Enzymatic degradation profile of the fibrin/PVA scaffolds over a period of 1, 3, and 5-day incubation in 0.25% Trypsin-EDTA. The fibrin/PVA scaffolds were cross-linked by EDC/NHS overnight or different concentrations of glutaraldehyde (GLA), including 0%, 0.05%, 0.2%, 0.5%, and 1%, respectively. Data represent mean ± SD, *n* = 3.

In order to evaluate the degradation profile of the fibrin/PVA scaffolds with different cross-linking degrees, trypsin was applied as an enzymatic proteolysis assay to compare the degradation of each scaffold over a relatively short period. FNG1PVA0.5 was selected as the formulation for making the scaffold and studying the effects of cross-linking methods (EDC/NHS vs. Glutaraldehyde) and different cross-linking degrees by glutaraldehyde on the degradation profile. The fraction of the remaining mass compared to the original mass for each sample was summarized in Figure 4.

Firstly, it was found that FNG1PVA0.5 without cross-linking (un-crosslinked) was completely decomposed in one hour, which can be attributed to the non-covalent interactions in the native fibrin filament structure between fibrin and PVA [47]. Secondly, the enzymatic stability of scaffolds corresponds with a cross-linking degree for the scaffolds which were cross-linked by different concentrations of glutaraldehyde (0.05%, 0.2%, 0.5%, and 1%); that is, the higher the cross-linking degree, the higher the stability against enzymatic proteolysis. This accords with the understanding that crosslinking can reinforce the stability of giant molecular matrices to resist degradation against enzymatic digestion [48]. Moreover, EDC/NHS-crosslinked FNG1PVA0.5 degraded completely after about 12 h. However, those that were cross-linked by glutaraldehyde were more stable compared with the EDC/NHS-crosslinked ones. This is also consistent with the finding of others that EDC/NHS-crosslinking confers less stability than glutaraldehyde-crosslinking for porous scaffolds [49]. Importantly, the incorporation of PVA can significantly improve the enzymatic stability of fibrin scaffolds. The pure fibrin scaffolds FNG1 + 0.2% GLA degraded completely after 1 day, whereas FNG1PVA0.5 + 0.2% GLA scaffolds took 5 days for complete degradation. The degradation behavior of biomaterial scaffolds is a key factor for the success of skin tissue regeneration. Since a too-fast degradation of the skin scaffold cannot provide effective protection of the wound site and need to replace the scaffolds frequently, causing inconvenience to the patients as well as an increase in cost. Therefore, the introduction of PVA in the FNG1PVA0.5 scaffold showed excellent superiority in improving the degradation stability. Moreover, increasing the concentration of glutaraldehyde for the cross-linking reaction resulted in a corresponding increase in the stability of FNG1PVA0.5 scaffolds.

### 3.4. Cytocompatibility of Scaffolds

To evaluate the cytocompatibility of the fibrin/PVA scaffolds, a quantified cell viability assay was performed by using the cell proliferation reagent kit-8 (CCK-8).

**Figure 5:**
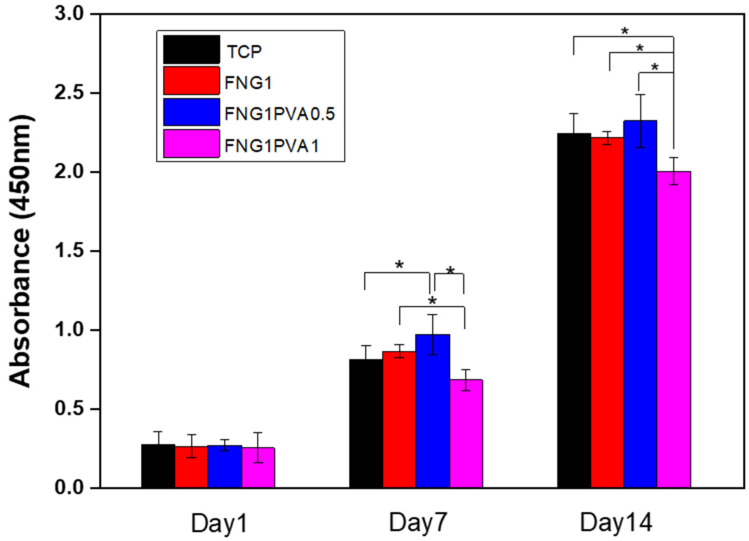
MSC proliferation TCP, FNG1, FNG1PVA0.5, and FNG1PVA1 scaffolds after 1, 7, and 14-day incubation analyzed by cell counting kit-8 (CCK-8) test. Data represent mean ± SD, *n* = 4, * *p* < 0.05.

Figure 5 shows MSC proliferation on the scaffolds on days 1, 7, and 14, measured by the CCK-8 assay, which is indicative of the quantity of metabolically active cells in the scaffolds. The data show that MSCs proliferate at similar rates as on TCP (tissue culture plastic) substratum on all the scaffolds, with a 2.6 times increase in cell number from day 1 to day 7 and a 4.0 times increase from day 7 to day 14. In addition, scaffolds formed at a lower ratio of PVA/FNG (FNG1PVA0.5) resulted in a slightly higher MSC cell attachment and growth, evident on day 7. This might be related to the narrower distribution of pore diameters of this scaffold maintained at the lower PVA incorporation. It has been proposed that scaffolds with higher interconnected porous structures can promote cell ingress, attachment, and proliferation [50]. Overall, the MSC proliferation data demonstrate favorable cytocompatibility of the scaffolds for cell growth and proliferation.

**Figure 6:**
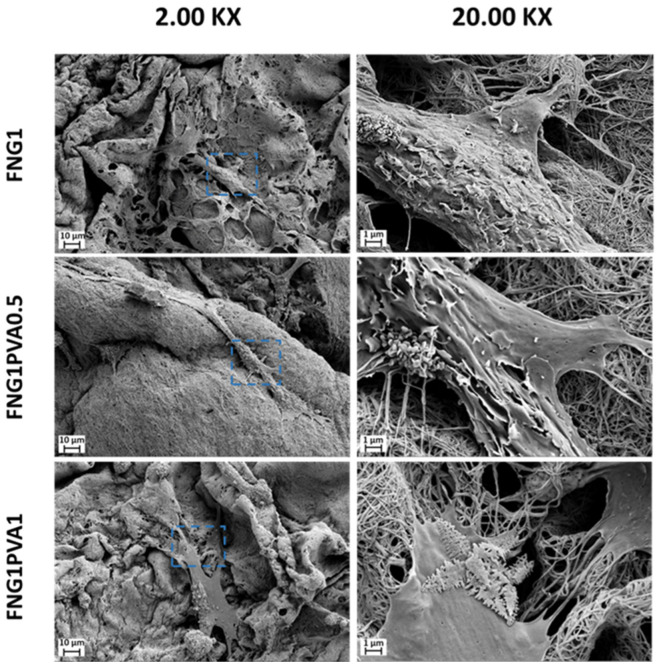
SEM images of MSCs on FNG1, FNG1PVA0.5, and FNG1PVA1 scaffolds after culturing for 14 days. The left column represents the micrographs taken at the magnification of 2.00 K×, while the right column represents the amplifying micrographs of the selected regions (blue dot boxes) in the corresponding left column at the magnification of 20.00 K×.

Figure 6 illustrates the MSC morphology after culturing on fibrin/PVA scaffolds for 14 days by SEM analysis. It can be seen that MSCs adhered on all scaffolds tightly with elongated and stretched morphology. Furthermore, cells penetrated into the scaffolds and extended multiple filopodial protrusions into the nano-fibrous structure of the scaffold. This further demonstrates the scaffolds’ cytocompatibility and support of cell-adhesive interactions.

### 3.5. The In Vivo Wound Healing Efficacy of the Fibrin/PVA Scaffolds

**Figure 7:**
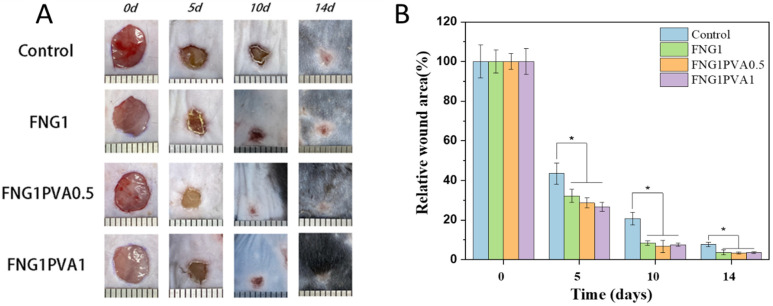
(**A**) Digital photographs of C57 mice wounds covered by fibrin/PVA scaffolds with different compositions at days 0, 5, 10, and 14. (**B**) Normalized wound area with control and different fibrin/PVA scaffolds treated wounds at days 0, 5, 10, and 14. Data represent mean ± SD, *n* = 5, * *p* < 0.05.

After making a full-thickness skin defect on the dorsal of the C57 mice, the wounds were covered with fibrin/PVA scaffolds with different compositions to evaluate their wound healing efficacy. Figure 7A shows the representative optical images of gross wound contraction in the different groups on day 0, day 5, day 10, and day 14, a combined effect of “purse-string” contraction of the reticular dermis and inward epithelial migration. All wounds were characterized by an initial defect of around 6 mm in diameter, which then decreased over time. It was found that the fibrin and fibrin/PVA scaffolds showed significantly faster wound recovery than the control group. To quantify the wound healing speed, the wound area was analyzed by Image J software, and the results were presented in Figure 7B. On day 5, the fibrin/PVA scaffolds-treated groups showed remaining areas of 28% and 26% for FNG1PVA0.5 and FNG1PVA1, respectively, while the value was 43% for the control group. A similar trend was observed on day 10, showing significantly reduced remaining areas on all fibrin and fibrin/PVA-treated wounds in comparison to the control group. Furthermore, the fibrin and fibrin/PVA-treated wounds had an unclosed wound area of only 3% vs. 8% in the control group by day 14. Overall, the fibrin/PVA scaffolds proposed here exhibited excellent wound-healing properties.

**Figure 8:**
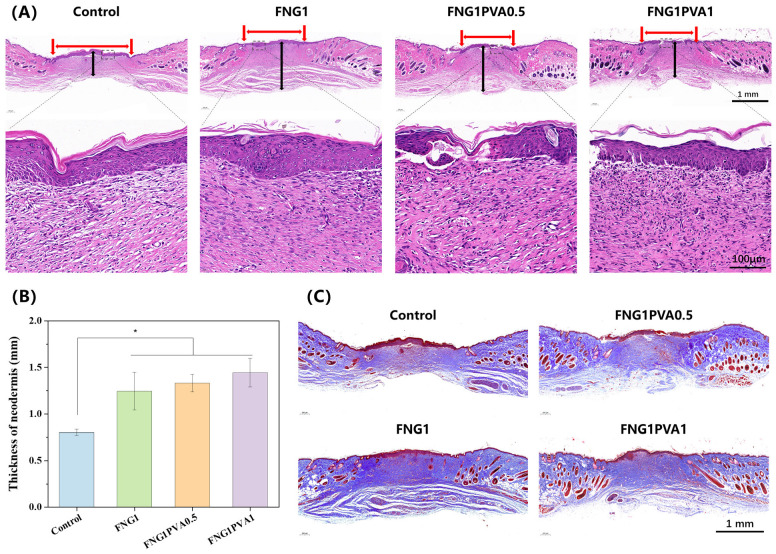
Histological staining images of regenerated skin tissue at the wound site on day 14. (**A**) H&E staining images. Red arrow: dermal space length. Black arrow: granulation tissue. (Scale bar = 1 mm/100 μm). (**B**) Quantified thickness of neodermis calculated from images of H&E staining. (**C**) Masson’s trichrome staining images (Scale bar = 1 mm). Data represent mean ± SD, *n* = 3, * *p* < 0.05.

**Figure 9:**
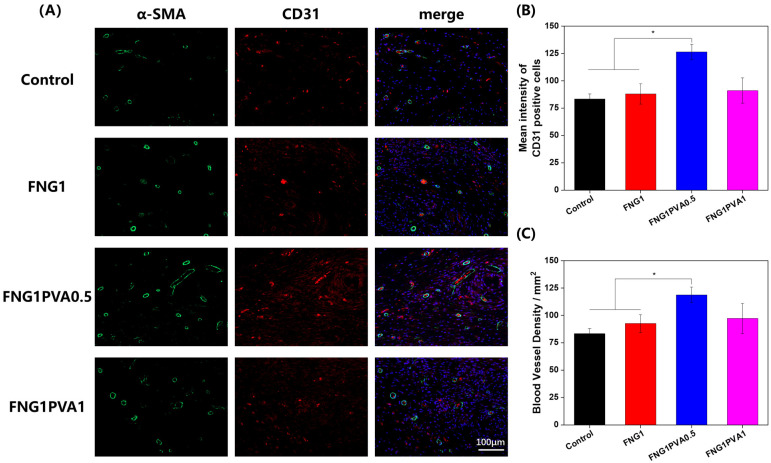
Immunofluorescence analysis of regenerated skin tissue at the wound site on day 14. (**A**) Representative staining images of CD31 (red), α-SMA (green), and nuclei (DAPI, blue); scale bar: 100 μm. (**B**) Quantitative intensity of CD31 positive cells. (**C**) Quantitative graph of blood vessel density on day 14 corresponding to α-SMA-positive staining in diabetic wounds. Data represent mean ± SD, *n* = 3, * *p* < 0.05.

In order to further analyze the in vivo biocompatibility and therapeutic effects at the histological level, H&E (Figure 8A), Masson trichrome staining (Figure 8C), and immunostaining (Figure 9) were conducted on the regenerated skin tissues collected on day 14. Significantly, although purse-string wound contraction is apparent in all groups, the healing process involves the extent of neodermal tissue formation and epithelial overgrowth of this neodermis. As can be seen in Figure 8A, the H&E-stained section demonstrates restoration of the panniculus carnosus layer in the FGN/PVA scaffold-engrafted wounds. In addition, the wound areas on the dorsal of mice have all epithelialized on day 14 in the control and fibrin/PVA-scaffold-treated groups. However, in the control group, it was found that the epithelium did not fuse with the basement membrane continuously, indicating that the re-epithelialization was not complete, which corroborated the overall wound closure data (Figure 7). In addition, the dermal diameter length (red arrows) was the longest in the control group. By contrast, the re-epithelialization was more advanced and mature in fibrin/PVA scaffold groups, evidenced by the complete formation of the stratified dermis, a fusion of epithelium with the basement membrane, and a much shorter dermal space length. More importantly, the larger magnification of histological analysis indicates that there is no obvious infiltration of inflammatory cells on the scaffold-treated wounds, demonstrating the intrinsic biocompatibility of the scaffolds. Furthermore, the quantified thickness of neodermis formed from granulation tissue (black arrows) was summarized in Figure 8B. A markedly deeper thickness was found in the fibrin and FGN/PVA scaffolds-treated groups than in the untreated control group, meaning the wounds healed very well. The Masson’s trichrome staining corroborates the results of the H&E, showing that the fibrin/PVA scaffolds-treated wounds mediated much more collagen fiber formation compared to the control group.

A well-recognized difference in skin healing between mice and humans is the much greater degree of wound contraction in mice, with humans having greater hypodermis containing cellular reservoirs of MSC and endothelial cells. These translate into differences in scaffold function between species. Our results indicate that the contraction of the scaffold by the surrounding skin and ingrown granulation tissue, more evident with the more stable FGN/PVA composites, can present an issue in the murine model which may not arise in human clinical application. Bearing this in mind and taking the results together, the faster wound healing speed, the more complete re-epithelialization with much thicker granulation tissue, as well as the higher collagen fiber deposition in the fibrin/PVA-scaffold-treated wounds demonstrated the excellent therapeutic effects of the scaffolds as skin regeneration materials.

Angiogenesis plays an important role in the wound-healing process; thus, the effects of scaffolds on angiogenesis were visualized by immunofluorescent staining of CD31 and α-smooth muscle cells (α-SMA) (Figure 9A), and the quantified values are shown in Figure 9B,C. Here, CD31 is the most common marker to represent the vascular endothelial cells, while α-SMA is used to show the vascular wall. As shown in Figure 9A, it was found that the untreated control group exhibited a low level of positive staining. By contrast, wounds in fibrin and fibrin/PVA-treated groups displayed significantly increased positive staining of both CD31 and α-SMA in the neodermis layer at day 14. These results suggested that the fibrin and fibrin/PVA scaffolds possess pro-angiogenic properties, which are likely to contribute to the tissue reconstruction and healing process in the full-thickness skin excision-defect model.

## 4. Conclusions

In summary, we have presented an emulsion templating method to fabricate novel fibrin/PVA scaffolds for potential application as a skin substitute. Through the characterization of scaffold structure, pore size, mechanical properties, and degradation profile, as well as cytocompatibility, it was found that FNG1PVA0.5 with a lower ratio of PVA/FNG was the optimized formulation for scaffold fabrication in terms of better interconnected porous structure, improved mechanical properties, and enhanced proteolytic stability. Notably, FT-IR data illustrated the formation of a new hydrogen bond between FNG and PVA after the incorporation of PVA in the composite scaffold. Furthermore, these physico-chemical properties exert important roles in cellular behavior. Thereupon, the FNG1PVA0.5 scaffold showed support for MSC ingress, penetration, and proliferation within the scaffold over a 14-day period similar to that shown on tissue culture plastic. Cross-linking allows for independent control of proteolytic degradation to give a broad stability range suitable for clinical application. The stability achieved with 0.2% glutaraldehyde is sufficient to allow for histological integration, which supports and accelerates organized granulation and supports efficient migration of the epithelial wound boundary over the formed neodermis.

Importantly, the in vivo wound healing results illustrate that the PVA/FNG scaffolds could significantly accelerate wound healing, accompanied by greater vascularisation, deeper neodermal tissue and more collagen fiber deposition, and faster re-epithelial overgrowth. Notably, the histological analysis demonstrates complete integration of the scaffolds to form organized neodermal tissue without pro-inflammatory reaction within the 14-day study period. The immunofluorescent staining of CD31 and α-SMA results indicate the pro-angiogenic effects of the PVA/FNG scaffolds. Overall, our results demonstrate that the novel FNG1PVA0.5 scaffold proposed here has promising properties for skin tissue engineering to treat patients with a large area of skin loss or chronic wounds caused by such conditions as burn and diabetic foot.

## Data Availability

The data that support the findings of this study are available on request from the corresponding author.
